# Pulmonary infection of Schizophyllum commune diagnosed by metagenomic next- generation sequencing: A case report

**DOI:** 10.1097/MD.0000000000031465

**Published:** 2023-03-17

**Authors:** Jing Zhang, Guoliang Xu, Jiumei Shen, Gangfu Ye

**Affiliations:** a The Second School of Clinical Medicine, Guangzhou University of Chinese Medicine, Guangzhou, China; b Department of Respiratory and Critical Care Medicine, Xiamen Hospital of Chinese Medicine, Xiamen, China; c Department of Pathology, Xiamen Hospital of Chinese Medicine, Xiamen, China.

**Keywords:** fungal infection, metagenomic next-generation sequencing (mNGS), Schizophyllum commune

## Abstract

Fungal infection is common and difficult to be diagnosed timely in clinical, for its various kinds and similar manifestations. The rare pulmonary fungal infection such as Schizophyllum commune was one of the harder ones and misdiagnosed in usual.

**Patient concerns:**

We report a 32-year-old female which was diagnosed with Metagenomic Next-Generation Sequencing (mNGS). She was hospitalized with the complaint of 4 months and more of repeated cough and expectorating. The chest computer tomography revealed left lower lobe pathological changes, but antibiotics were ineffective. No positive results were found in laboratory tests, including sputum culture and the pathology of lung puncture biopsy.

**Diagnoses:**

mNGS of lung biopsy was performed and detected the sequence number of Schizophyllum for 11.

**Interventions:**

The patient was treated with voriconazole and itraconazole successively.

**Outcomes:**

She recovered to health. There was no recurrence during follow-up.

**Lessons:**

mNGS as a diagnostic method could quickly detect pathogens through the processing of fragment, synthesis, comparison, and analysis of sample genes. It is suitable for detecting especially rare and polymicrobial infections. To our best knowledge, infection of Schizophyllum commune have not been reported in English literature with diagnostic method of mNGS.

## 1. Introduction

With increasing numbers of patients needing intensive care or who are immunosuppressed, mycoses caused by rare moulds become the focus of attention. Schizophyllum commune is one of this rare mold infections.^[1]^ It belongs to the Phylum Basiaiomycota, Subphylum Agaricomycotina, Order Agaricales which contains fungi, colloquially known as mushrooms and is widely distributed in nature, infects human by inhalation.^[2]^ For most clinical institution, the biggest problems lie in identification of Schizophyllum commune and Aspergillus in morphology by conventional laboratory. However, metagenomic next-generation sequencing (mNGS), as a diagnostic method, could quickly detect pathogens through the processing of fragment, synthesis, comparison and analysis of sample genes. Herein, we report a 32-year-old female with pulmonary infection of Schizophyllum commune diagnosed by mNGS, which accepted a tortuous but ultimately successful treatment for reference.

## 2. Case presentation

On August 27, 2019, a 32-year-old female, which had a long-term residence in Xiamen, China, was hospitalized with the complaint of “4 months and more of repeated cough and expectorating.” Accompanying symptoms included a large amount of yellow phlegm, nasal congestion and yellow runny nose. She had a history of pulmonary tuberculosis 12 years ago, nasal polyps operation which caused massive bleeding 10 years ago. She was also a case of paranasal sinusitis seeking medical advice in the Department of Ear-Nose-Throat, and born with lack of intelligence. Family history: her mother is mentally retarded and has died; her father has a history of facial hemangioma, which causing right face and lip deformity; her little brother is healthy. She lived in a low-income family with financial difficulties. In the past 4 months, she had some Chinese medicines and the symptoms repeated. Physical examination on admission: the body temperature was 36.5°C, blood pressure was 112/68 mm Hg, and her heart rate was 92 beats per minute. Auscultation did not reveal any abnormalities.

**Figure 1.**
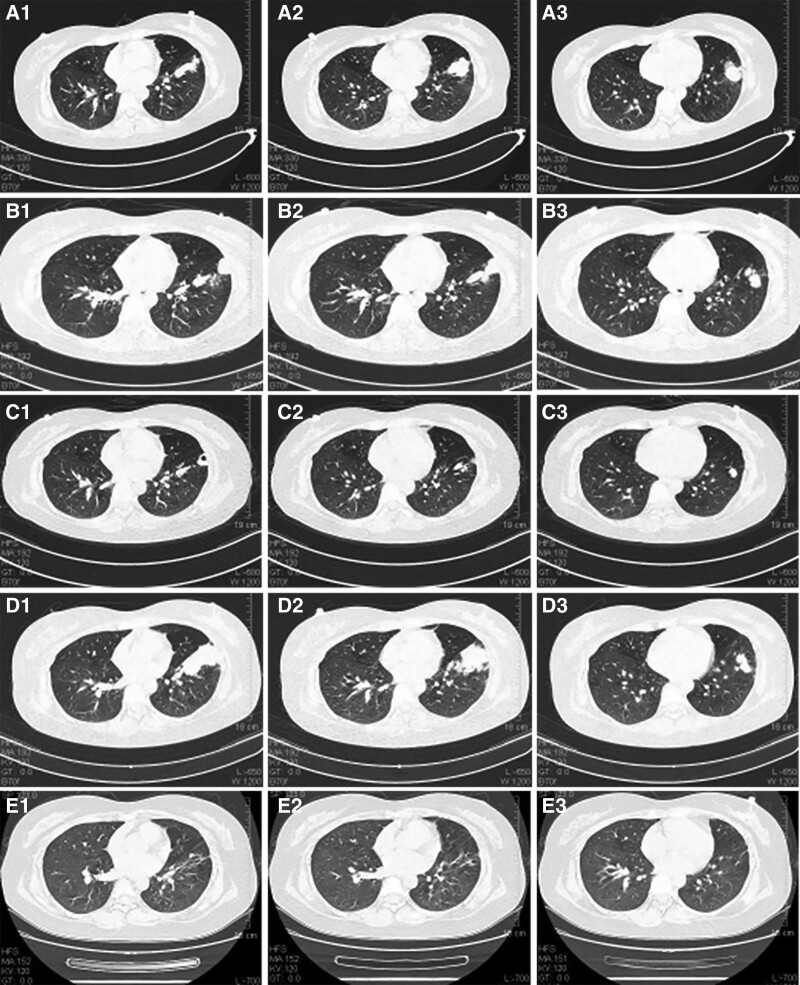
(A) Chest CT (2019-8-26) revealed left lower lobe pathological changes. (B) Chest CT (2019-10-4) showed progression of the lesion. (C) Chest CT (2020-1-2) showed that the exudation was obviously absorbed and cavity formed compared with the previous 1. (D) Chest CT (2020-5-19) showed progression of the lesion. (E) Chest CT (2020-10-27) showed that the focus of infection had been obviously absorbed. CT = computer tomography.

Laboratory tests on admission revealed a white blood cell count of 8.6 × 10^9^/L, eosinophils count of 0.7 × 10^9^/L, proportion of eosinophils for 7.8%, and C-reactive protein of 2.0 mg/L. Procalcitonin, biochemical, coagulation and defecation tests were normal. The result of blood GM test (0.69μg/L), G test (79.53 pg/mL), IgE (>2500 iu/mL) came out subsequently. Autoantinuclear antibody spectrum and anti-neutrophil cytoplasmic antibodies were negative. Antiacid bacilli in sputum was negative. At the same time no positive results were found in the sputum culture for 3 times. The chest computed tomography (CT) scan on admission was showed in Figure 1A. After 14 days treatment of antibiotics, the chest CT revealed no improvement and her family members asked to leave the hospital because they could not accept the puncture biopsy recommended by doctor.

**Figure 2.**
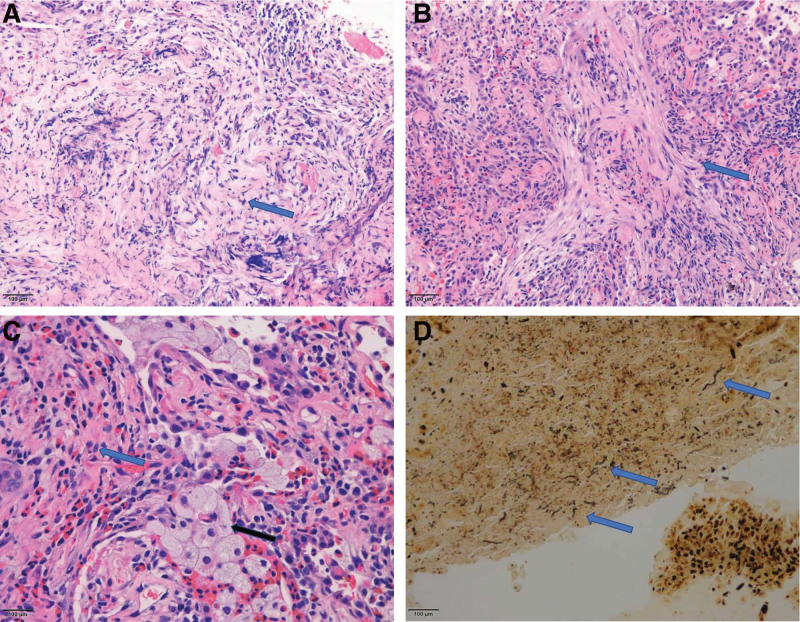
(A and B) The pathology showed fleshy change (bule arrow), HE × 200. (C) The pathology showed fleshy change (blue arrow) and foam cells (black arrow), HE × 400. (D) Fungal hyphae was found with method of special straining, W–S × 400.

About 1 month after discharge, the patient was hospitalized secondly due to aggravation of symptoms and advancing of chest CT (Fig. 1B). Then CT guided percutaneous lung puncture biopsy was performed on October 9, 2019. Pathology (Fig. 2A and B) showed coagulative necrosis, fleshy and fibrous changes, which indicated special infections, but could not be further typed. Tissue culture and all Special staining results were negative. This time, mNGS was taken into consideration and within 48 hours the result reported that the sequence number of Schizophyllum was 11 and no other pathogen was detected. We gave a diagnosis of pulmonary infection of Schizophyllum commune. Combined with the results of previous in vitro drug sensitivity test, voriconazole was given orally. After 2 months, Chest CT (Fig. 1C) showed better, however liver function showed worse. Considering voriconazole-related liver injury, we made a decision of drug withdrawal and protective treatment. Although the liver function gradually recovered, patient’s father refused to use antifungal drugs anymore because of the fear of liver injury.

In May 2020 (4 months after stopping voriconazole), the patient was admitted to the hospital fourthly due to the aggravation of cough and expectoration. Laboratory tests showed similarity with first hospitalization. The chest CT (Fig. 1D) was advanced compared with 4 months before. Bronchoscopic biopsy pathology and bronchoscopic alveolar lavage fluid culture still indicated fungal infection (Fig. 2C and D) but could not be further typed. Based on above evidence, pulmonary infection of Schizophyllum commune was considered and itraconazole was given orally. On October 27, 2020, the patient came up with good state and nowhere uncomfortable. Her chest CT (Fig.1E) showed the focus of infection had been obviously absorbed. Itraconazole was stopped in November. The patient was followed up in October 2021, and there was no recurrence.

## 3. Discussion

Schizophyllum is common in the environment, especially in the rotten wood, and can infect humans by inhalation. It often causes colonization, which then leads to a series of immune reactions, but not typical. The first case of Schizophyllum infection was reported in 1950.^[3]^ Since then, it has attracted more and more attention.^[2,3]^Its common infectious sites include skin, mucous membrane, sinuses, lungs, brain. Literature reports on Schizophyllum commune have mostly implicated the fungus with pulmonary manifestations ranging from sinusitis, allergic bronchopulmonary mycoses, brochial mucoid impaction, pulmonary fungal balls, asthma, chronic eosinophilic pneumonia, brochogenous cysts, and so on.^[4]^ Pulmonary imaging and clinical laboratory examination usually lack specificity. According to the vitro post infection experiment of mice,^[5]^ the mycology and laboratory research progress of Schizophyllum commune speculated that the pathogenesis may induce serious local inflammatory reaction and produces inflammatory suppurative granulomatous lesions and fibrosis. Morphologically, Schizophyllum commune with haploid strain cannot be distinguished from Aspergillus and other fungi with transparent separated hyphae.^[6]^ In addition, most clinical laboratories do not have the equipment and conditions for identification, so it often leads to missed diagnosis or misdiagnosis as Aspergillus.

mNGS is a rapid method to detect pathogen through gene fragmentation, synthesis and comparison. It is playing an important role in rare and severe infections in clinic.^[7]^ On the 1 hand, conventional microbial culture requires pathogens to be living, corresponding growth conditions, long culture time and demanding, while morphological identification needs to provide special equipment, and laboratory physician need to have the ability to identify rare pathogens, which is obviously difficult to achieve. On the other hand, compared with the polymerase chain reaction, the huge shared gene information database of mNGS can provide rich comparative sources and reduce the difficulty of diagnosis of rare infections. The feature of no need for specific primers makes it easier to find unknown pathogens. The high-throughput method shortens the time of detection and is conducive to the rapid and accurate diagnosis of pathogenic microbes in patients with severe infection. At the same time, it also has the characteristics of high sensitivity and wide coverage. Although there are still some problems need to be solved in mNGS, such as inconsistent operation standards and result interpretation, hardness of process quality control, we could overcome by analysis of clinical manifestations. For this case, the sinusitis, jelly like sputum, chest CT bronchial mucus thrombus, lump like changes, blood eosinophil count and increased IgE, combined with cellulose like exudation and purulent inflammatory changes in lung biopsy pathology, fungi were found in the culture of bronchoscopic alveolar lavage fluid for many times, all indicated fungal infection. Finally, percutaneous lung biopsy was sent to mNGS for detecting, which plays a vital role in the determination of diagnosis.

## 4. Conclusions

Diagnostic method as mNGS could quickly detect pathogens and effectively complements the shortcomings of delayed diagnosis and low positive rate of conventional microbial morphological identification. It is suitable for detecting especially rare and polymicrobial infections. Meanwhile, it should be noted that whether the pathogen obtained from mNGS is colonization or invasion needs further comprehensive analysis by clinicians according to specific clinical characteristics.^[8]^This case indicated that it is necessary to prompt diagnostic procedures and standardization.

## Author contributions

**Conceptualization:** Jing Zhang, Gangfu Ye.

**Data curation:** Jing Zhang, Jiumei Shen.

**Formal analysis:** Jing Zhang, Guoliang Xu, Gangfu Ye.

**Investigation:** Jiumei Shen, Gangfu Ye.

**Methodology:** Jiumei Shen.

**Supervision:** Guoliang Xu, Gangfu Ye.

**Validation:** Guoliang Xu.

**Writing – original draft:** Jing Zhang, Jiumei Shen.

**Writing – review & editing:** Jing Zhang, Guoliang Xu, Gangfu Ye.
